# Consequences of eye fluke infection on anti-predator behaviours in invasive round gobies in Kalmar Sound

**DOI:** 10.1007/s00436-017-5439-5

**Published:** 2017-04-06

**Authors:** Henrik Flink, Jane W. Behrens, P. Andreas Svensson

**Affiliations:** 1 Department of Biology and Environmental Science, Linnaeus University, 39182 Kalmar, Sweden; 2 National Institute of Aquatic Resources (DTU Aqua), Technical University of Denmark, Charlottenlund, Denmark

**Keywords:** Biological invasion, Parasitism, PITT, Trematoda, Anti-predator behaviour

## Abstract

Larvae of the eye fluke, *Diplostomum*, emerge from snails and infect fish by penetrating skin or gills, then move to the lens where they may impair the vision of the fish. For the fluke to reproduce, a bird must eat the infected fish, and it has been suggested that they therefore actively manipulate the fish’s behaviour to increase the risk of predation. We found that round gobies *Neogobius melanostomus*, a species that was recently introduced to the Kalmar Sound of the Baltic Sea, had an eye fluke prevalence of 90–100%. We investigated how the infection related to behavioural variation in round gobies. Our results showed that the more intense the parasite-induced cataract, the weaker the host’s response was to simulated avian attack. The eye flukes did not impair other potentially important anti-predator behaviours, such as shelter use, boldness and the preference for shade. Our results are in accordance with the suggestion that parasites induce changes in host behaviour that will facilitate transfer to their final host.

## Introduction

By causing sublethal fitness costs as well as mortality, parasites act as one of the most important selective agents in organisms and shape both evolutionary and ecological processes (Lefèvre et al. 2009; Rohr et al. 2009; Schmid-Hempel 2009; Kekäläinen et al. 2014). Parasites may also modify host’s appearance and manipulate their behaviour (Poulin 1994). Indeed, the behaviour of parasitised animals can be explained as a mixed phenotype, because they often represent a composite of characters originating from both host and parasite genotype (Dawkins 1982; Barber et al. 2000). Expression of mixed phenotypes is particularly common in animals infected by parasites that are transmitted trophically, that is, parasites that require one host to be eaten by another to complete their life cycle (Barber et al. 2000; Poulin 2010). Such parasites can benefit from an increased rate of transmission by manipulating behaviours of their intermediate host so that it becomes more susceptible to predation by the parasite’s definitive host (Poulin 1994). It is expected that selection for such host manipulation, referred to as parasite increased trophic transmission (PITT), is strong (Lafferty 1999; Barber et al. 2000; but see Cézilly et al. 2010). If host manipulation results in high probability of predation also by predators not suitable as hosts, the parasite may however fail to improve its transmission rate. This predicts that host manipulation should evolve towards suppression of the risk of being predated by non-hosts relative to host predators (Barber et al. 2000). Despite ample indirect evidence of PITT in a variety of parasitised organisms, there is an on-going debate over how important of a process host manipulation is (e.g. Poulin 1994, 1995, 2010; Cézilly et al. 2010; Hafer and Milinski 2015). In fact, altered behaviours in parasitised animals may rather result from competition between parasite and host interests or they may be a mere side effect from pathology (Poulin 1994, 2010; Barber et al. 2000).

Parasites can play a major role in biological invasions (reviewed in Prenter et al. 2004). For example, exposure to parasites from the newly colonised range can cause disadvantages to the invader and may explain spatial and temporal variation in invasion success (Pizzatto et al. 2012). Populations of established introduced species occasionally collapse, known as boom and bust, and such collapses may be ascribed to pathogens or parasites native to the colonised area (Simberloff and Gibbons 2004).

Since 1990, the invasive round goby *Neogobius melanostomus* (Pallas, 1814), native to the Ponto-Caspian region, has expanded its range and increased in abundance all over the Baltic Sea and the Great Lakes (Sapota and Skóra 2005; Azour et al. 2015; Ojaveer et al. 2015). In the Baltic Sea, it now dominates the catch in various coastal fisheries, and it is frequently found in offshore catches (Ojaveer et al. 2015). Round gobies are like most fishes infected by numerous different parasites both in their native and invasive areas (Kvach et al. 2014). However, to what extent this may affect their behaviour and fitness remains unknown.

The trematode genus *Diplostomum* contains several common parasites infecting the eyes of freshwater and brackish water fish and thus they are called eye flukes (Chappell 1995; Gibson 1996). The complex life cycle of *Diplostomum* spp. start with free-swimming miracidiae that in order to reproduce asexually infect aquatic snails (Chappell 1995; Seppälä 2011). Cercariae burst out from snails in multitudes and when they find a fish, their second intermediate host, they infect it through the skin or gills. Within 24 h, the cercariae have migrated to the eye where they develop to metacercariae (Chappell 1995). Eye flukes are most commonly found in fish lenses, but also in the retina or the vitreous humour (Gibson 1996). To reach the next stage in the life cycle, infected fish needs to be eaten by a bird, which often is a gull, tern, goose or duck (Gibson 1996; Seppälä 2011). In the bird intestine, the parasite matures, reproduces sexually and releases its eggs together with the bird faeces.

Eye flukes are known to cause harmful effects to fish as they induce cataract, i.e. lens opacities, by mechanical destruction, metabolic excretions and by being partially or completely opaque (Shariff et al. 1980). High parasite burden in fish may lead to blindness, emaciation and even death (Shariff et al. 1980; Chappell 1995; Karvonen et al. 2004). Recent molecular studies show that it is common for fish to be infected with more than one species of eye fluke, and that different fish species harbour different parasite communities in their lenses (Rellstab et al. 2011). In dace *Leuciscus leuciscus* (Linnaeus, 1758), three-spined stickleback *Gasterosteus aculeatus* (Linnaeus, 1758) and Arctic charr *Salvelinus alpinus* (Linnaeus, 1758), impaired vision due to eye fluke infection has been shown to reduce feeding capability, thereby increase feeding times and predation risk (Crowden and Broom 1980; Owen et al. 1993; Voutilainen et al. 2008). Also, infected dace spend more time near the water surface where they are exposed to avian predation (Crowden and Broom 1980) and infected rainbow trout *Oncorhynchus mykiss* (Walbaum, 1792) have a reduced escape response towards aerial attacks and thus a suspected increase in susceptibility to predation by birds (Seppälä et al. 2004, 2005b, 2012). How much any of this applies to the round goby remains unknown.

*Diplostomum* is often the parasite taxa with highest prevalence in round gobies (e.g. Muzzall et al. 1995; Kvach and Skóra 2007; Kvach and Stepien 2008; Francová et al. 2011). However, the prevalence varies considerably, from 0% in some populations to prevalence’s towards 90% in others (Muzzall et al. 1995; Camp et al. 1999; Kvach et al. 2014). Parasite intensity (i.e. number of eye flukes per infected fish) varies similarly and can locally be very high (Kvach and Skóra 2007; Kvach and Winkler 2011; Kvach et al. 2014). In the present study locations, southeast of Sweden in the Kalmar Sound, the invasive round goby was first observed in 2013 and has since then become one of the most dominant fish species (Nilsson 2014, 2016). Compared to other species of fish, a considerable proportion of round gobies caught at the study location in 2015 and 2016 had intense eye fluke infection (H. Flink and D. Amnebrink, unpublished data). If eye fluke-infected round gobies suffer some of the same effects as described above for other fish hosts, it may affect the potential for the continued invasion of this species. In localities with high infection intensity, such as in the present study location, we hypothesise that eye flukes have harmful effects on their host as seen in other species, for example by impairing vision and thus anti-predator behaviours. We tested this by investigating if the intensity of infection was related to the expression of round goby anti-predator behaviours. Furthermore, we measured prevalence and intensity of eye fluke infections, and carried out molecular species determination of eye flukes.

## Method

### Collection and husbandry

The study was performed at Kalmar Sound Laboratory, Kalmar, Sweden. Two field collections were made using a seine net in shallow water (0–1 m). In October 2015, 88 adult round gobies (average total length ± S.D. 9.2 ± 1.5 cm) were caught for the behavioural experiments in two bays in Kalmar sound (Kattrumpan 56° 39′ 56″ N, 16° 22′ 25″ E and Tallhagen 56° 41′ 6″ N, 16° 22′ 9″ E). To get a second estimate of eye fluke prevalence and intensity in the field, 50 more adult round gobies were collected at Kattrumpan in April 2016.

The fish collected in October 2015 were held in stock tanks with continuously flowing brackish water (7 ± 0.2 psu) until January 2016, 1 month before behavioural experiments began. The fish were then transferred to holding aquaria (50–60 L) with dechlorinated tap water and commercial sea salt (SERA marine sea salt, Germany) at a salinity of 7.0 psu, corresponding to the salinity at the location of collection. Four fish were housed in each 40-L aquarium and five fish in each 50-L aquarium. The holding aquaria had individual filters and partial water changes were conducted every second week. All tanks were provided with coarse gravel and plastic tubes (14 cm by Ø 4.5 cm) as shelters. The light regime was set to mimic daily changes in the outdoors diurnal light cycle and was changed continuously during the holding period. The water temperature varied between 13 and 15 °C in a temperature-controlled room. The fish were fed small pieces of herring and blue mussels ad lib three times per week. During the time in these tanks, infected fish fed readily of the provided food, they appeared healthy (except for eye fluke-induced cataract) and behaved normally.

### Eye dissection

Directly following the behavioural trials (October 2015 sample) or maximally 1 week after capture (April 2016 sample), individual fish was euthanised using an overdose of benzocaine. Total length and wet weight was measured to the closest millimetre and milligram, respectively. The cataract score (degree of parasite-induced cataract coverage) was assessed in the fish lenses with a direct ophthalmoscope (HEINE Beta 200S LED, USA). Cataract score was determined with a subjective scale as used in earlier studies (Karvonen et al. 2004; Seppälä et al. 2005ab, 2008, 2011, 2012; Karvonen and Seppälä 2008), where 1 = no cataracts, 2 = less than 50% coverage, 3 = more than 50% coverage and 4 = 100% coverage or completely white lens. Individual cataract intensity was quantified as the average score from both eyes, allowing for comparison with previous studies (e.g. Seppälä et al. 2005b, 2011; Karvonen and Seppälä 2008). Petri dishes and microscope slides were prepared with saline solution, each eye was removed and put on an individual dish, and the lenses were excised and placed on a slide. All metacercariae from each fish, both from the lens and outside the lens, regardless of developmental stage, were counted with a dissecting microscope. The exact site of parasites found outside the lens (i.e. in the vitreous humour or in tissues under the retina) could not be determined. All flukes found were identified as the genus *Diplostomum* by morphological characters (Key: Gibson 1996). Metacercariae were put aside in saline solution for further morphological and molecular determination.

### Molecular analysis of metacercariae

Metacercariae from 32 randomly selected round gobies (October 2015 sample) were sampled for molecular species determination. Most parasites were found in the lens of the eye and most were translucent; however, there were also some opaque parasites and some that were found in other areas of the eye. Two parasites from each of the 32 fish were selected at random from the lens, and if the fish had parasites from other areas of the eye and/or that were opaque, these were also sampled. In total, 87 metacercariae were sampled: 64 randomly sampled and in addition 23 that were opaque and/or found outside the lens. Before molecular analysis, the metacercariae were fixed in boiling water for 1 min and individual parasites put on a microscope slide and photographed through a light microscope to enable morphological comparisons. The parasite was placed in ethanol for 2 to 3 weeks prior to DNA extraction.

DNA was extracted from the metacercariae using the QIAamp DNA Mini Kit. PCR (T100 Thermal Cycler Bio-Rad, CA, USA) was used to amplify sequences containing the ribosomal internal transcribed spacer (ITS) region. The PCR reactions contained 5.0 μL DNA extraction, 2.5 μL PCR buffer (15 mM MgCl_2_), 0.2 μL Taq polymerase (5.0 U/μL), 0.1 μL dNTP (10 mM) and 0.1 μL of each primer, in a total volume of 25 μL. As forward and reverse primers, D1 (Galazzo et al. 2002) that targets the 3′end of the 18S gene and BD2 (Luton et al., 1992) that targets the 5′end of the 28S gene were used, respectively. The PCR configuration was as follows: first, an initial denaturing step at 95 °C for 5 min, then 35 cycles of denaturation at 95 °C for 30 s, annealing at 53 °C for 30 s, elongation at 72 °C for 60 s and a final elongation at 72 °C for 7 min. To verify the PCR-product, samples were run through electrophoresis (1.6% agarose gel containing 0.01% SYBR Safe stain) and visualised with UV light. Due to methodological difficulties, only 42 out of 87 metacercariae were successfully amplified. Successful amplifications were purified by using polyethylene glycol (PEG) and ethanol precipitation, and sent for sequencing to Eurofins Genomics, Ebersberg, Germany. Sanger sequencing was performed with the 3730XL DNA analyser (Applied Biosystems, MA, USA). As forward and reverse sequencing primers, BD1 (Luton et al. 1992) and BD2 were used, respectively. BD1 targets the 3′end of the 18S gene. Out of 42 sequences, only 32 had sufficient quality, therefore the rest was omitted from the analysis.

A molecular phylogenetic analysis was performed on 32 metacercariae sequences collected from 21 round gobies. In addition, 22 ITS1 sequences of *Diplostomum* spp. were used as reference, and two sequences of the Diplostomidae species *Tylodelphys scheuringi* (Hughes, 1929) and *Alaria taxideae* (Swanson and Erickson 1946) were used as out-group (method adapted from Haarder et al. 2013). Sequences were aligned using ClustalW version 2.1 (Larkin et al. 2007) as implemented in Geneious version 9.1.3 (Kearse et al. 2012) and the phylogenetic analysis was conducted in MEGA7 (Kumar et al. 2016). The analysis was run with the maximum likelihood method based on Kimura 2 + G (Kimura 1980), as suggested as the best-fit model in MEGA7, and a bootstrap consensus tree was inferred from 1000 replicates.

### Light avoidance

**Fig. 1:**
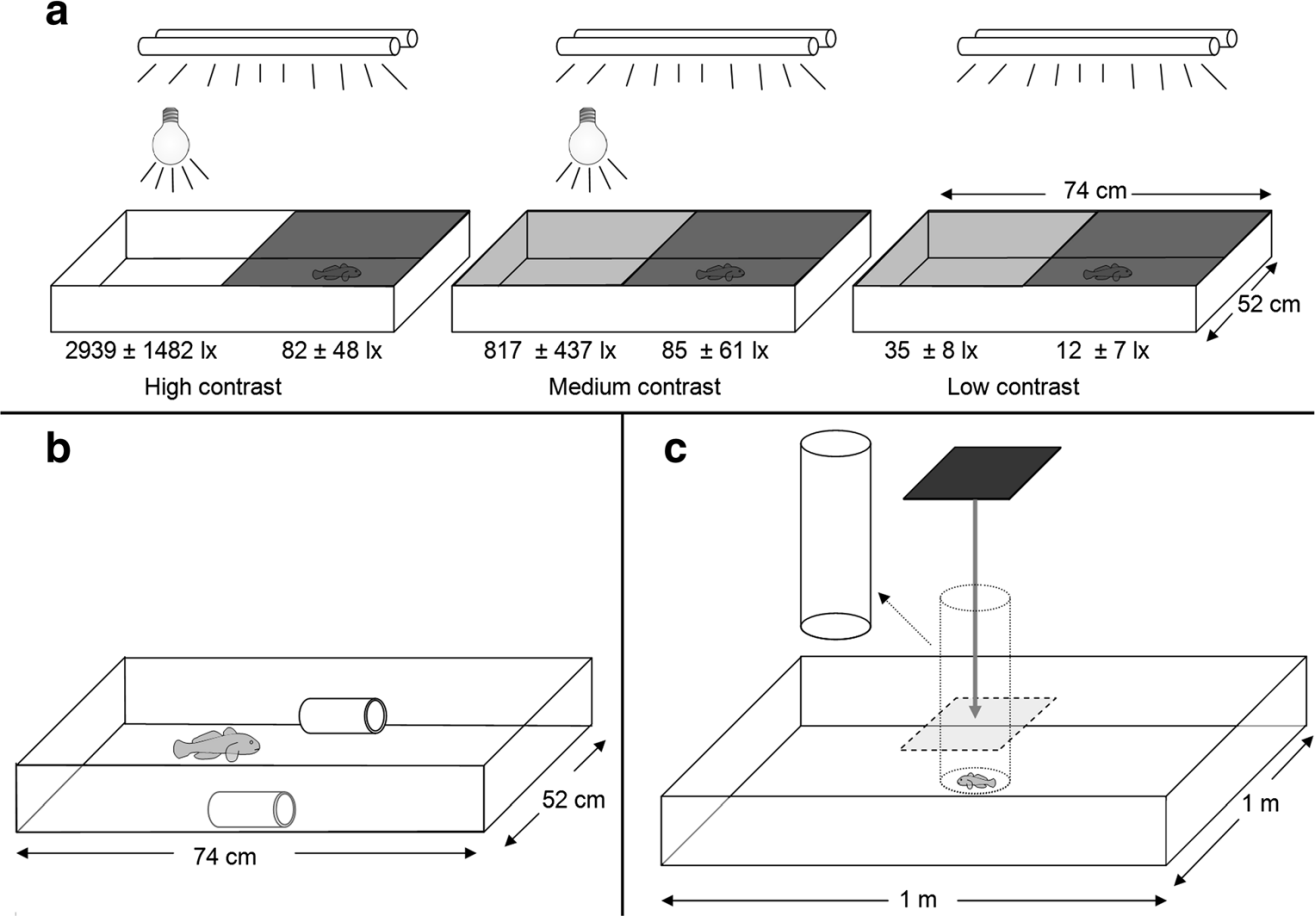
Test arenas used to quantify anti-predator behaviours in round gobies. In the light avoidance test (**a**), three different contrast settings were created using combinations of overhead fluorescent lights and shade films. An additional light was placed above the brighter part of the arena in the high and medium contrast levels. *Numbers* indicate mean illuminance in lux ± S.D. In the sheltering and boldness test (**b**), two shelters were placed along the sides of the arena. Escape behaviour (**c**) was quantified by allowing gobies to settle inside a clear vertical tube in the centre of the arena (*dotted line*). After removing the tube, an aerial attack was simulated by dropping a black square to immediately above the fish (*dashed line*). See text for details

To shelter during daylight is a typical behaviour of round gobies (Dubs and Corkum 1996; Borcherding et al. 2013; Capelle et al. 2015) and thus we expect healthy gobies to avoid strong light and prefer shaded areas. We investigated the relationship between eye fluke infection and the preference for shade (*N* = 88). The experimental arenas measured 74 × 52 cm (length × width) with a water depth of 10 cm. To limit the fish from burrowing, the arenas had only 1 cm layer of fine sand. One half of the aquaria were shaded to a greater extent than the other by using 0, 1 or 2-layers of window film 25% visible light transmittance (BILTEMA, Sweden) that were placed on the glass sheets covering the tank (Fig. 1a). The test was carried out at three different light intensity levels. In the high and medium contrast setting, a 250 W metal halide lamp (OSRAM HQI-E 250W/D, Germany) was placed above the aquaria in addition to the fluorescent lights that were for the low contrast setting. The light difference in illuminance between light and shade was approximately 35× (high contrast), 10× (medium contrast) and 3× (low contrast). Individual round gobies were netted from its holding aquarium and placed on the border between the bright and the dark half 5–6 h before the trial. IR-sensitive video cameras above the aquaria were used to study the position of the fish. During 2 h, between 8:30 and 10:30 pm, the location of the fish was recorded and the time spent in the dark versus the bright area was quantified to the nearest second. The proportion of time spent in the darker half was then used to estimate the preference for shade.

### Shelter use and boldness

Chasing and catching fish with dip-net is a simplified method of simulating predator attack and has previously been used to evaluate vulnerability to predation, for example in eye fluke-infected rainbow trout (Seppälä et al. 2004, 2005b; Gopko et al. 2015). To determine if the round gobies differed in their ability to find and use shelter, 61 fish from the light avoidance test were also screened for shelter usage and boldness. The same arenas were used as in the light avoidance test, but without the overhead lamp and the glass covers. Two plastic tubes (14 cm by Ø 4.5 cm, same type as in the holding tanks) were added to the longer sides of each aquarium (Fig. 1b). The fish were then slowly but continuously chased with a rectangular dip-net (16 cm × 13 cm) until it found shelter in one of the tubes. The complete trial was video-recorded and the time taken from the onset of chasing until the fish was completely inside the tube was subsequently quantified to the nearest second and used as an estimate of ability to find shelter. When the fish had entered the shelter, it was left alone. The time from this point and until the fish had emerged from the tube with its entire body was used as an estimate of boldness, however, allowing the fish a maximum of 30 min to emerge from the shelter. Boldness is a trait with high repeatability in individual round gobies (*R* = 0.77, Flink and Svensson, unpublished data) and is often evaluated in studies of animal personalities (Sih et al. 2004).

### Response to simulated aerial attack

In order to study how cataract may affect escape response following aerial attack, the fish previous screened for shelter use and boldness (*N* = 61) were exposed to a simulated aerial predator. The fish were placed in a new experimental arena, measuring 100 × 100 cm (Fig. 1c). The water depth was 10 cm and the water was replaced every fourth trial. The bottom of the arena was white and no shelter was provided to create an area perceived as risky in terms of exposure to aerial predation. The fish was put in a transparent vertical cylinder (Ø 15 cm) in the centre of the aquarium. After giving the fish 10 min to settle, the cylinder was gently lifted out of the way with a pulley system. Five seconds later, a black square (25 × 22 cm) was released from above the fish and fell 78 cm before stopping immediately above the fish, 1 cm above the water surface. The escape response was observed and was quantified as either 0 = no response/freezing, or 1 = immediate escape (i.e. dashing away from the centre). Other possible escape responses such as slow swimming, jumps and staggered dash (Barber et al. 2004) were not observed.

### Data analysis

The collected data were analysed with R version 3.2.2 (R Core Team 2015). If necessary to obtain normality of residuals, variables were log-transformed, and if parametric assumptions still were not satisfied, non-parametric tests were used. Binary response variables were analysed with generalised linear models (GLM) and depending on the degree of overdispersion, either binomial or quasibinomial errors were used. Boldness, measured as in time to emerge from shelter, was analysed with time-to-event analysis using a Cox proportional hazards regression model in the Survival package (Thernau 2013). This allowed us to account for censoring in the data (i.e. not all fish emerged during the trial). Body condition was calculated by taking the residuals from a mass/length log-log linear regression of all fish caught at the same time (October 2015 sample: *N* = 88, *R* ^2^ = 0.98; April 2016 sample: *N* = 50, *R* ^2^ = 0.98). When calculating the total number of metacercariae per fish, the sum of parasites in both eyes was used. However, in four fish, the total number of parasites was extrapolated from one eye due to unsuccessful quantification of metacercariae in the other eye. In the analysis of behavioural experiments we omitted, two fish with both their lenses erupted.

## Results

### Assessment of cataract and dissection

**Fig. 2:**
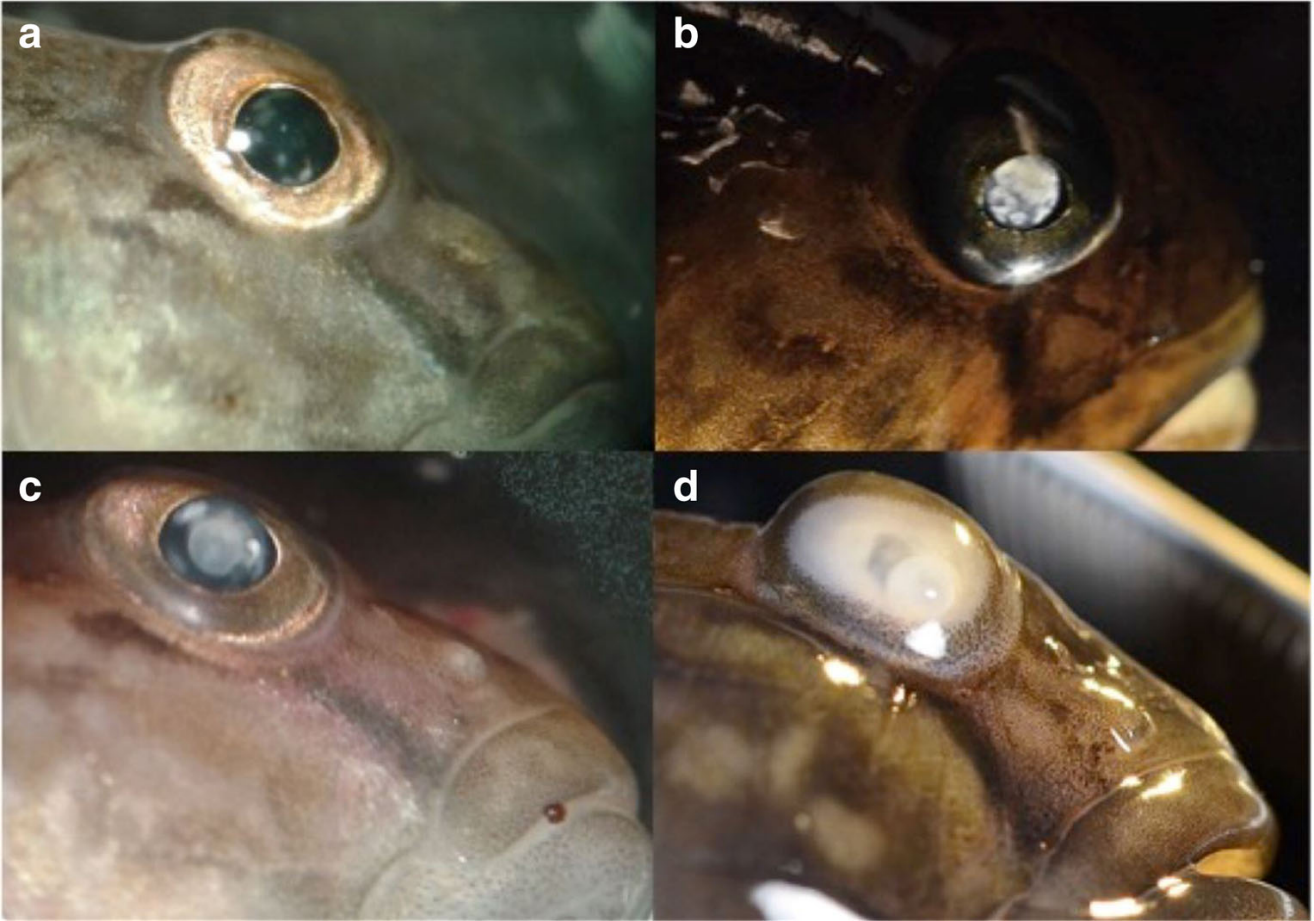
Infected round gobies from the October 2015 sample. Cataract intensity was assessed with an ophthalmoscope. Most commonly, fish had less than 50% cataract coverage (**a**); however, several fish had more than 50% coverage (**b**, **c**). A few fish had 100% coverage and in rare cases completely white lenses, sometimes dislocated (**d**) or completely erupted

**Fig. 3:**
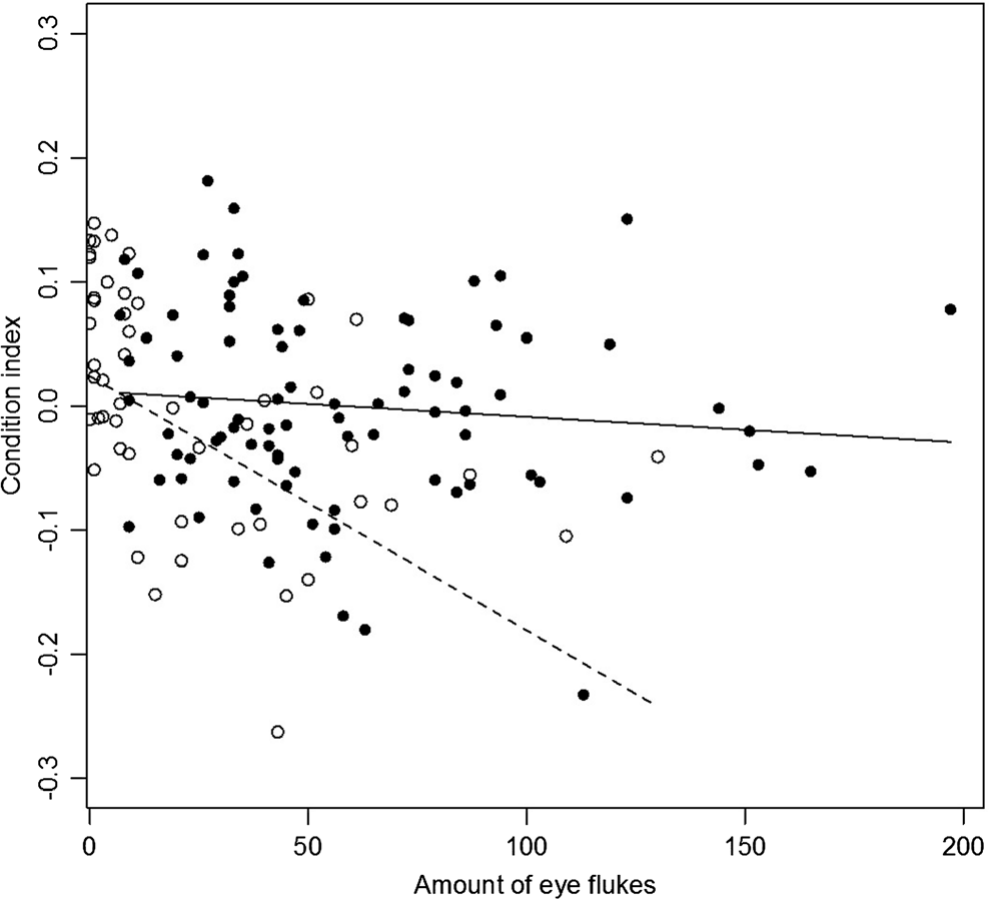
The body condition of fish with high parasite load was lower than average in field (April 2016 sample: *white dots*, *dashed line*). There was no such correlation in fish that had been held in lab for 4 months (October 2015 sample: *black dots*, *solid line*)

The prevalence of *Diplostomum* spp. in round gobies collected in October 2015 was 98% (86 infected fish out of 88). Two fish did not have any eye flukes but had erupted lenses on both eyes. Because it is known that severe infections may lead to destruction of the lenses (Shariff et al. 1980), it is likely that these fish had been infected earlier. One more fish had an erupted lens, while the other lens was intact but with a high intensity of eye flukes. All fish had detectable eye fluke-induced cataract, and in 35% of fish, the cataract coverage was more than 50% in both eyes (mean cataract score ≥3, Fig. 2). The average intensity (± standard deviation) of metacercariae per fish was 58 ± 39 (*N* = 86). As expected, fish with higher parasite load had higher cataract score (Spearman’s rank correlation: *N* = 55, *ρ* = 0.43, *p* < 0.001). However, there was no correlation between the total amount of eye flukes and the condition of the fish (Spearman’s rank correlation: *N* = 86, *ρ* = 0.14, *p* = 0.20, Fig. 3), or between cataract score and fish condition (Spearman’s rank correlation: *N* = 56, *ρ* = 0.16, *p* = 0.24). It should be noted that these fish had been fed ad lib for 4 months in the lab prior to experiments. Larger fish had higher load of eye flukes (Log-log linear regression model, *N* = 86, *R* ^2^ = 0.27, *p* < 0.001). In total, 4722 eye flukes were found and according to their morphology, all flukes were fully developed metacercariae and infective for birds (see Sweeting 1974). Seven metacercariae were found outside the lens in six different fish.

A second sample (*N* = 50) was collected in April 2016 in order to compare prevalence between newly caught fish with that of the lab-housed fish. Prevalence in this sample was 90%, which is not significantly different from the first sample (Fisher’s exact test, *p* = 0.80). However, the mean intensity, 27 ± 30 (*N* = 45), was lower than in the first collection (Wilcoxon rank sum test *W* = 882.5, *p* < 0.001). There was no correlation between the length of the fish and the amount of eye flukes (Spearman’s rank correlation *N* = 50, *ρ* = 0.23, *p* = 0.12). There was however a negative association between the parasite load and the condition of the fish (Spearman’s rank correlation *N* = 50, *ρ* = 0.59, *p* < 0.001, Fig. 3). In the second sample, a total of 1201 eye flukes were found. A large number of these were immature. Only one eye fluke was found outside the lens.

### Molecular analysis of metacercariae

**Fig. 4:**
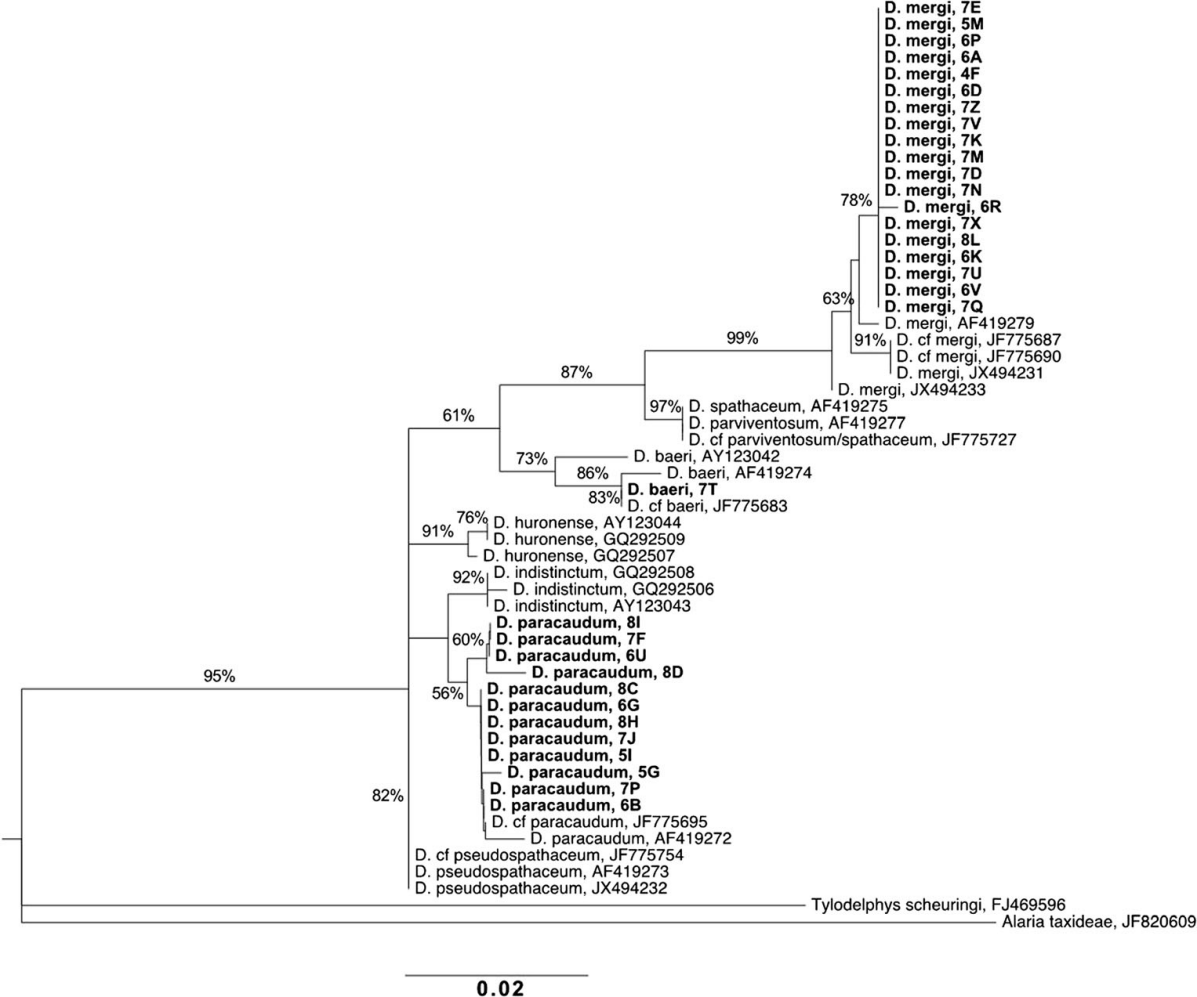
Molecular phylogenetic tree estimated with the maximum likelihood method based on the Kimura 2-parameter model of 54 sequences of *Diplostomum* spp. and two out-group sequences. Sequences of the 32 parasites isolated from round gobies (October 2015 sample) are emphasised in *bold*. The other 22 sequences are representatives for known Diplostomidae species (see Haarder et al. 2013 for selection criteria). The analysis shows that the eye flukes sampled in this study are branched in three clades. Bootstrap values above 50% are shown next to each branch. The tree is drawn in scale, with branch lengths measured in the number of substitutions per site

**Fig. 5:**
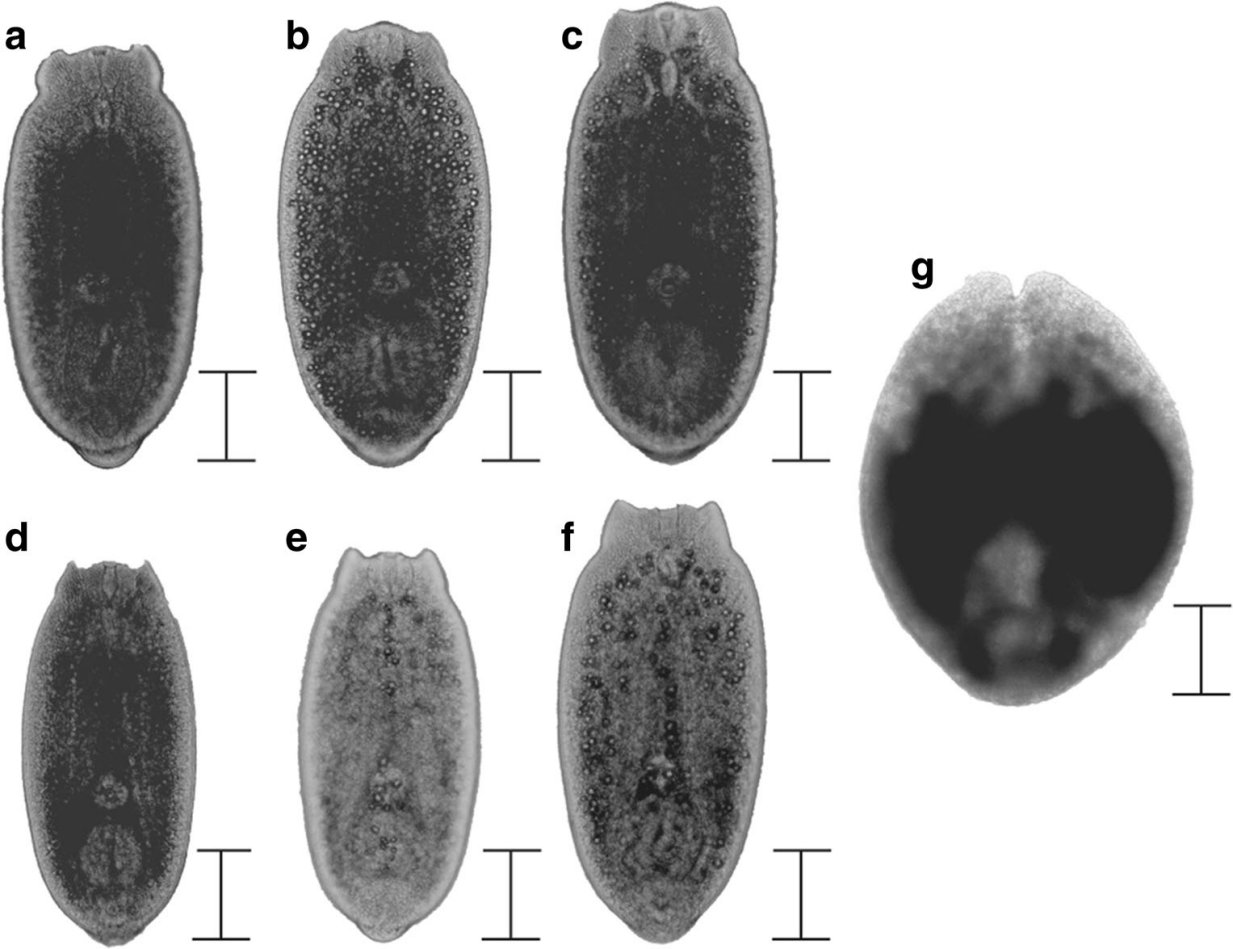
A selection of metacercariae from round goby lenses, determined by phylogenetic analysis as *D*. *mergi* (**a**–**c**) and *D*. *paracaudum* (**d**–**f**). Also, one specimen of *D*. *baeri* (**g**) found outside the lens. Parasites were fixed in boiling water and then photographed through a light microscope. *Scale bars* 0.1 mm

The phylogenetic analysis branched the isolated parasites into three different clades (Fig. 4). In total, 19 isolates were determined as *Diplostomum mergi* (Dubois, 1932), 12 as *Diplostomum paracaudum* (Iles, 1959) and one as *Diplostomum baeri* (Dubois, 1937) (see Fig. 5 for photographs). The *D*. *baeri* isolate was found outside the lens and was opaque. One isolate of *D*. *mergi* and one of *D*. *paracaudum* were also found outside the lens, whilst the others were found in the lens. In nine fish, successful sequencing was performed from more than one parasite. Out of these, five fish were infected with both *D*. *mergi* and *D*. *paracaudum*.

### Light avoidance

**Fig. 6:**
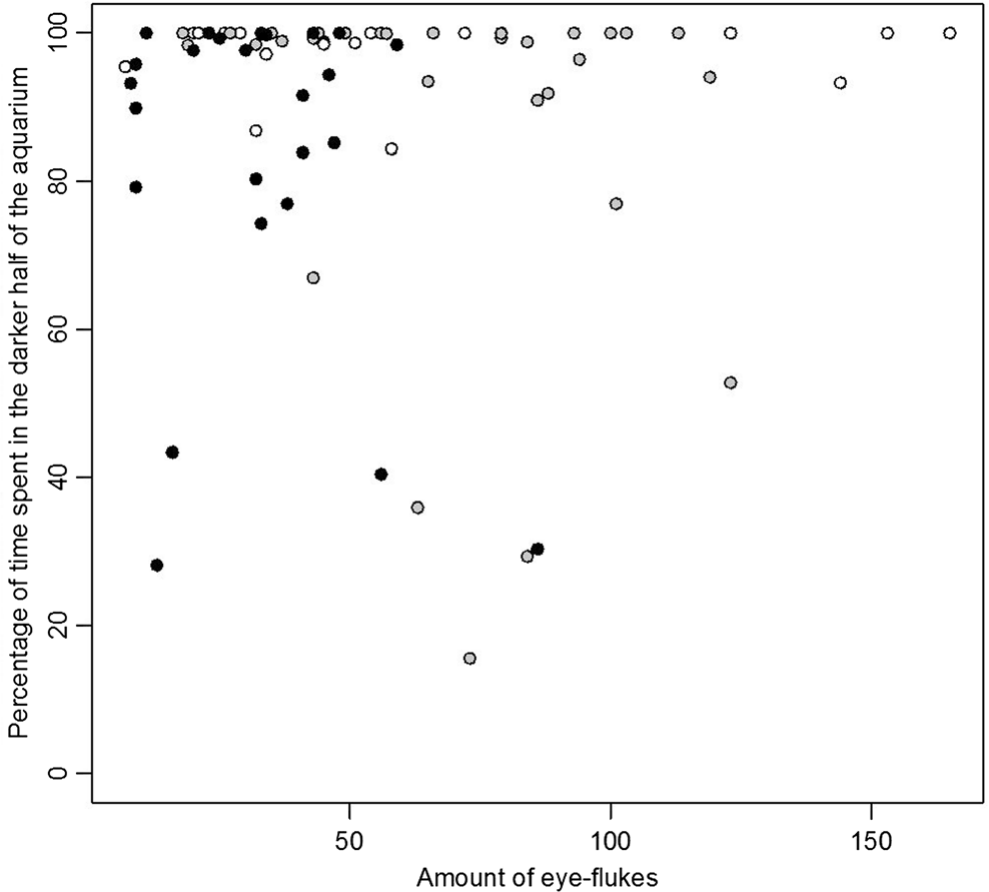
Percentage of time spent in the darker half of the aquarium during the three light intensity treatments in relation to the amount of eye flukes the round gobies harboured. Independent of treatment, the fish spent most of the time in the darker half. The different light intensity treatments are indicated as *circles* with *white* (high contrast), *grey* (medium contrast) and *black* background (low contrast)

The majority of fish were actively swimming during observations, and 71 out of 78 fish spent more than 50% of the time in the dark half (Wilcoxon signed rank test *V* = 46, *p* < 0.001, Fig. 6). This was true for all three settings, although in the lowest contrast setting fish spent relatively more time in the brighter area (Kruskal-Wallis rank sum test *χ* ^2^ = 8.91, *p* = 0.01). Cataract score was not correlated to the preference for shade in either fish from high (Spearman’s rank correlation *N* = 17, *ρ* = 0.24, *p* = 0.36) or medium contrast treatment (Spearman’s rank correlation *N* = 32, *ρ* = 0.14, *p* = 0.43). Cataract score was not assessed in fish from low contrast trials; however, the highly correlated measure parasite intensity was unrelated to shade preference (Spearman’s rank correlation *N* = 26, *ρ* = 0.04, *p* = 0.84).

### Shelter use and boldness

All the tested fish found shelter with a maximum time of 102 s (mean 26 ± 23). There was no significant relationship between cataract score and the time it took to reach shelter (GLM with Gamma errors *N* = 53, *t* = 1.18, *p* = 0.25). The fish that did not emerge from the shelter within the maximum time (23 of 57 fish) obtained the ceiling value of 1800 s (30 min) and were treated as censored data points in this analysis. The average time the fish took to emerge from shelter was 1197 ± 608 s. Boldness was not associated with cataract score (Cox proportional hazards regression model *N* = 54, *z* = 1.04, *p* = 0.30). There was no association between the time it took to find shelter and the time to emerge from shelter (Cox proportional hazards regression model *N* = 56, z = 1.07, *p* = 0.29).

### Response to simulated aerial attack

**Fig. 7:**
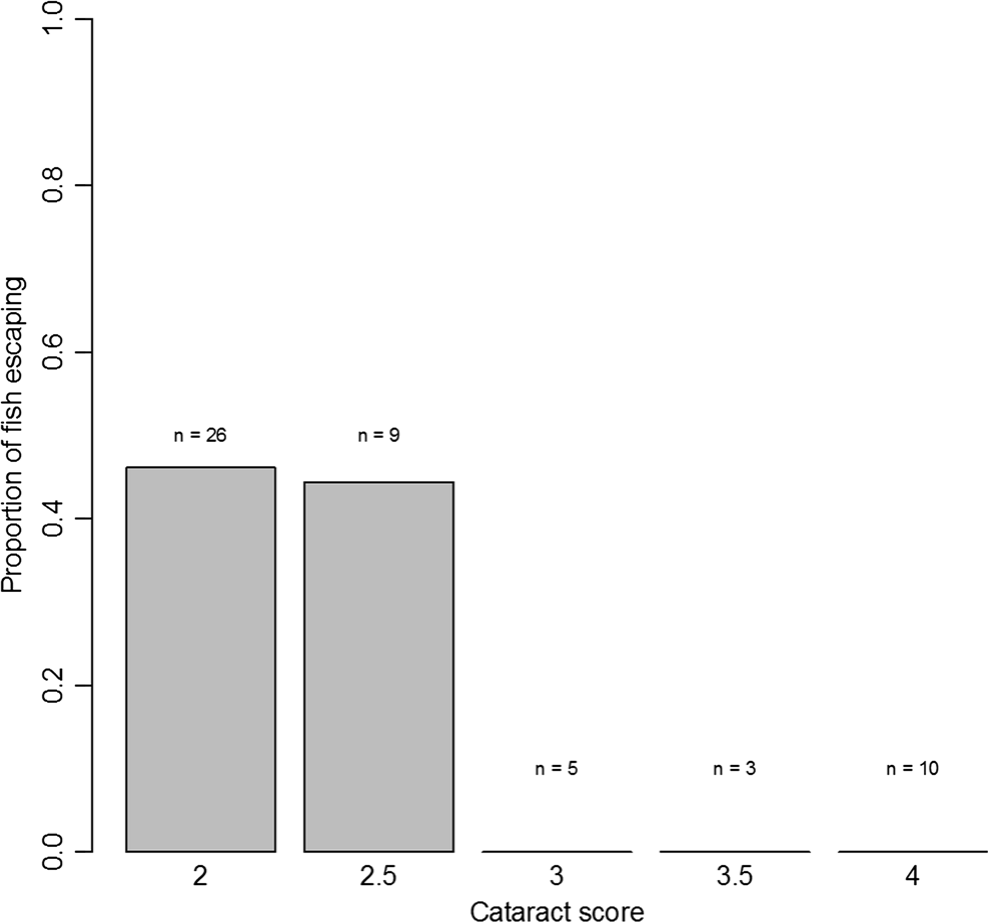
Round gobies were less likely to flee following a simulated aerial attack when cataract coverage was severe. No fish with more than 50% cataract coverage in both eyes responded by fleeing. The cataract score (average from both eyes) was calculated as follows: *1* = no cataracts, *2* = less than 50% coverage, *3* = more than 50% coverage and *4* = 100% coverage or completely white lens

The cataract score was negatively related to the propensity to escape following the simulated attack, that is, fish with severe cataract fled less frequently (GLM with quasibinomial errors *N* = 53, *t* = 2.50, *p* = 0.01, Fig. 7). None of the fish with >50% cataract coverage in both eyes (cataract score ≥3) fled.

## Discussion

Previous studies have proposed that eye flukes induce phenotypic changes that increase the susceptibility of fish hosts to predation (Crowden and Broom 1980; Owen et al. 1993; Seppälä et al. 2004, 2005a, b, 2008; Voutilainen et al. 2008) and that this effect is caused by impaired vision of the fish (Seppälä et al. 2005b, 2012). It has also been proposed that the increased susceptibility to predation is specifically targeting avian predators, suggesting it is an adaptation to increase the likelihood of the suitable final host eating the fish (Seppälä et al. 2006a, 2012). Our results are in accordance with these notions, suggesting that round gobies with severe eye fluke infection have reduced escape response to simulated aerial attack while more general anti-predator behaviours appear unaffected.

Fish clearly avoided the brighter parts of the aquarium irrespectively of degree of cataract. Such a preference was expected from round gobies if they perceive the darker part as less risky in terms of exposure to visual predators, and, clearly, the parasite did not change the individual fish’s ability to respond to differences in light intensity. Eye fluke-induced cataract has been suggested to impair nocturnal behaviour of Arctic charr, increasing their susceptibility to visual predators (Voutilainen et al. 2008). In addition, rainbow trout with cataract has reduced preference of dark background colouration, where the fish are less conspicuous, and impaired ability to adjust colouration to the background (Seppälä et al. 2005a). Our results demonstrate that round gobies with intense cataract could assess the present light intensity differences, including a relatively low contrast. However, it remains unknown whether round goby nocturnal behaviours are affected by cataract. The severity of the parasite-induced cataract was neither associated with the individuals’ ability to find shelter when chased nor with their boldness. This is in agreement with a study on rainbow trout where time spent in shelter, general activity level, and reaction to a simulated fish attack was unaffected by fluke infection and cataract (Seppälä et al. 2012). They suggested that host manipulation by eye flukes is driven by impaired vision and that fish vision is crucial to detect and avoid aerial predators, whereas eye fluke infection has no or little effect on other senses, such as the lateral line, which can be used to avoid for example predatory fish (Seppälä et al. 2012).

The observed reduced escape response following an aerial attack in fish with severe cataract suggests that this behavioural change is indeed caused by parasites, and likely by impairing the vision. This is in agreement with previous results in rainbow trout, where infected fish exhibit reduced escape response to aerial attacks (Seppälä et al. 2004, 2005b). However, as also shown by Seppälä et al. (2005b), the effect was observable first when cataract coverage had exceeded 50%. Thus, when cataract formation is intense, eye fluke infection appears to change host escape response in a way that increases susceptibility to predatory birds (Seppälä et al. 2004, 2005b, 2008). Yet, despite the consensus between these laboratory-derived results, the only available field study found that susceptibility to bird predation was not affected by eye flukes in rainbow trout (Seppälä et al. 2006b).

The molecular species determination showed that the parasite isolates from infected round gobies in the Kalmar Sound branched within clades of three different *Diplostomum* species, namely *D*. *mergi*, *D*. *paracaudum* and *D*. *baeri*. However, the small sample size may have led to an underestimation of the actual number of *Diplostomum* species. Eye fluke species typically have a low specificity in what fish species they infect as intermediate host (Karvonen et al. 2006) and fish are often infected by multiple fluke species at the same time (Rellstab et al. 2011 and references therein). Our molecular analysis suggests that *D*. *mergi* and *D*. *paracaudum* often coinfects round gobies. All three species are well known to the Baltic region (Rellstab et al. 2011), thus it is unlikely that these parasites were introduced together with the round goby when it first was transported to the Baltic Sea. *D*. *baeri* is known to reside in the vitreous humour of the eye (Rellstab et al. 2011), and the only isolate we found of this species was situated outside the lens. The molecular species determination is not absolutely certain without other kinds of data, such as morphometric measurements, and should be interpreted with caution (Cavaleiro et al. 2012).

The field prevalence of eye flukes in the round gobies caught in Kalmar Sound in this study were remarkably high at 90–100%, yet still comparable to prevalence’s reported in round gobies from both the Great Lakes of North America (Muzzall et al. 1995) and the Dnieper delta in the Black Sea, Ukraine (Kvach et al. 2014). Eye fluke prevalence and intensity are known to change seasonally, with the highest levels during the warmer part of the year (Mehrdana et al. 2015), and this may be one explanation for the small but significant difference in intensity between our two sampling occasions. It is common that *Diplostomum* prevalence and intensity can become very high, at least locally. From the Bothnian bay, in the Baltic Sea, prevalences of 90–100% and intensities above 20 parasites per infected individual have been reported in several other fish species, such as dace, roach *Rutilus rutilus* (Linnaeus, 1758), and ruffe *Gymnocephalus cernua* (Linnaeus, 1758) (Seppälä et al. 2011). All fish caught in the October sample had detectable eye fluke-induced cataract and one third of the fish had more than 50% cataract coverage in both eyes. If these numbers are representative for the Kalmar Sound population, a large proportion of the fish would potentially have an increased susceptibility to predation by piscivorous birds. There may however be differences in eye fluke prevalence and intensity between different depths and habitats of Kalmar Sound and it is possible that the proportion of fish with severe cataract may be selectively removed by predators.

Only in the first sample did we find a correlation between the length of the fish and the amount of eye flukes. We expected this since infection of eye flukes is cumulative through the life of fish and longer fish are expected to be older (Wootten 1974; Owen et al. 1993). In the second sample, the fish were smaller and varied less in length, and there may thus have been insufficient variation for this effect to be detected. In contrast, a negative correlation between the eye fluke intensity and the condition of the fish was only found in the second sample, where fish were dissected shortly after capture. Since this relationship was not found in the fish that had been fed ad libitum for 4 months in the lab, a possible explanation is that infected fish in the field have an impaired feeding due to eye fluke-induced cataract, as observed in several other fish species (Crowden and Broom 1980; Owen et al. 1993; Voutilainen et al. 2008).

The most important avian predators of round gobies in the Baltic Sea are great cormorants *Phalacrocorax carbo* (Linnaeus, 1758) and grey herons *Ardea cinerea* (Linnaeus, 1758), and they may locally feed almost exclusively on round gobies (Jakubas 2004; Bzoma and Meissner 2005). Therefore, it has been suggested that these species can help reduce the population growth of the round gobies in the Baltic (Ojaveer et al. 2015). It is plausible that the impaired escape response of infected round gobies to aerial attacks observed in this study might increase susceptibility to predation by the great heron and other birds that feed by striking from above. Intense eye fluke infection may thus, potentially, help limit the severity of the ongoing round goby invasion of the Baltic Sea.

In conclusion, our results show that eye fluke infection is highly prevalent in round gobies from the present study location in the Baltic Sea, and that several native fluke species infect round gobies simultaneously. Our results also suggest that eye fluke infection causes no changes in shelter behaviour, boldness or preference for shade. However, intense parasite-induced cataract impairs the escape response to aerial attacks, which might increase susceptibility to bird predation. Intense infection is also correlated with poor condition of fish in field, possibly due to impaired feeding capability.
